# Urinary NGAL Outperforms ^99m^Tc-MAG3 Renography in Predicting DCD Kidney Graft Function

**DOI:** 10.3389/ti.2025.13818

**Published:** 2025-05-12

**Authors:** Esther N. M. de Rooij, Tirsa T. van Duijl, Ellen K. Hoogeveen, Fred P. H. T. M. Romijn, Friedo W. Dekker, Cees van Kooten, Christa M. Cobbaert, Johan W. de Fijter

**Affiliations:** ^1^ Department of Nephrology, Leiden University Medical Center, Leiden, Netherlands; ^2^ Department of Clinical Epidemiology, Leiden University Medical Center, Leiden, Netherlands; ^3^ Department of Clinical Chemistry and Laboratory Medicine, Leiden University Medical Center, Leiden, Netherlands; ^4^ Department of Nephrology, Jeroen Bosch Hospital, Den Bosch, Netherlands; ^5^ Department of Nephrology and Hypertension, Antwerp University Hospital, Antwerp, Belgium; ^6^ Laboratory of Experimental Medicine and Paediatrics (LEMP), University of Antwerp, Antwerp, Belgium

**Keywords:** DCD donation, NGAL, urinary biomarkers, delayed graft function (DGF), kidney transplantation

## Abstract

Recipients of donation after circulatory death (DCD) kidneys are at high risk for delayed graft function (DGF) due to severe ischemia-reperfusion injury. We compared urinary biomarkers in predicting the duration of DGF with the tubular function slope (TFS) as the gold standard. In 89 DCD kidney transplant recipients, urinary TIMP-2, IGFBP7, B2M, NGAL, KIM1, CXCL9, and UMOD were quantified by LC-MS/MS analysis on postoperative days (PODs) 1, 4 and 10. Interstitial fibrosis and tubular atrophy (IF/TA) were assessed with protocol biopsies at POD 10. TFS was calculated with ^99m^Tc-MAG3 renography. Predictive performance was compared with AUCs from ROC analyses. Of all 89 recipients, 22% experienced no (<7), 22% mild (≥7–14), 29% moderate (≥14-<21) and 26% severe (≥21 days) fDGF. The OR for the presence of IF/TA was 1.9 (95% CI:0.4; 10.0) for mild to moderate and 15.0 (95% CI:2.7; 84.8) for severe compared to no fDGF. At POD 4, urinary NGAL and fractional NGAL excretion (FE-NGAL) outperformed TFS and other biomarkers in predicting fDGF with AUCs of 0.97, 0.98 and 0.92, respectively. At POD10, FE-NGAL and PCR best predicted severe vs*.* mild to moderate fDGF, with AUCs of 0.74 and 0.76 versus 0.65 for TFS. Therefore, urinary NGAL and FE-NGAL may provide a viable alternative to ^99m^TcMAG3 renography for monitoring fDGF clearance or guiding kidney transplant biopsy to exclude additional acute rejection.

## Graphical Abstract

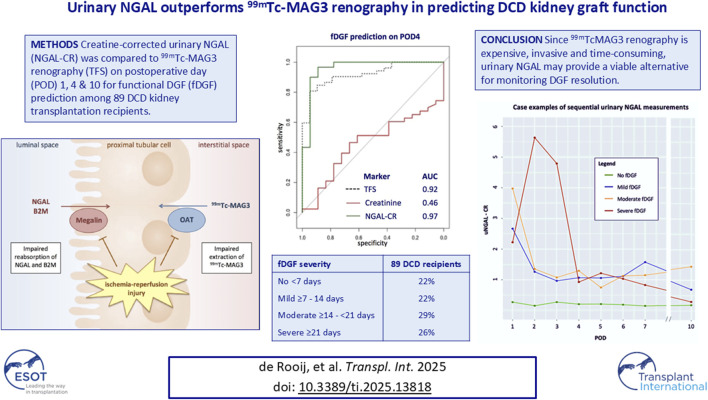

## Introduction

For the majority of patients with end-stage kidney disease (ESKD), kidney transplantation is the preferred modality of renal replacement therapy (RRT), but with the ongoing gap between supply and demand, the waiting time while on dialysis is increasing [1]. Owing to the shortage of kidneys available for transplantation, many countries use donation after circulatory death (DCD) kidneys to expand the potential donor pool [2]. In the Netherlands, from 2017 to 2021, the relative contribution of DCD increased from 56% to 66% of all deceased kidney transplants [3].

Kidneys from DCD donors have a higher risk of severe ischemia-reperfusion injury (IRI) compared to kidneys from donation after brain death (DBD) or those from living donors. The longer initial warm ischemia time (WIT) to which DCD kidneys are exposed increases the risk of primary nonfunction (PNF) and delayed graft function (DGF), with the latter estimated to be up to 50% [1, 2]. DGF is a manifestation of ischemia-reperfusion or acute kidney injury (AKI), most commonly due to acute tubular necrosis (ATN), which causes post-transplantation oliguria or anuria, increased allograft immunogenicity and may increase the risk of early acute rejection [3–6].

Traditionally, DGF has been defined as the need for dialysis in the first week after kidney transplantation [7]. However, since the indication for dialysis is clinically determined by nephrologists on an individual basis, this dialysis-based definition is subjective and does not always reflect the lack of adequate glomerular filtration. Therefore, the function-based definition of DGF (fDGF) has been proposed as an alternative for retrospective evaluation. fDGF is established when serum creatinine fails to decrease by at least 10% per day for 3 consecutive days within the first week after kidney transplantation [8].

Predicting the duration of fDGF and monitoring for the occurrence of a concomitant early acute rejection episode remains a major challenge in the first weeks after kidney transplantation [9]. Sequential ^99m^Technetium-mercaptoacetyltriglycine (^99m^Tc-MAG3) renography can be used to identify the evolution of tubular function in the case of DGF. ^99m^Tc-MAG3 renography allows for the calculation of a standardized tubular function slope (TFS), which reflects the ^99m^Tc-MAG3 uptake by renal tubular cells during the first minutes after ^99m^Tc-MAG3 injection [10, 11]. The TFS has previously been shown to be a sensitive biomarker of functioning proximal tubular epithelial cells (PTECs) and has been associated with fDGF and long-term graft function [10]. However, ^99m^Tc-MAG3 renography is an expensive, invasive and time-consuming investigation.

**FIGURE 1:**
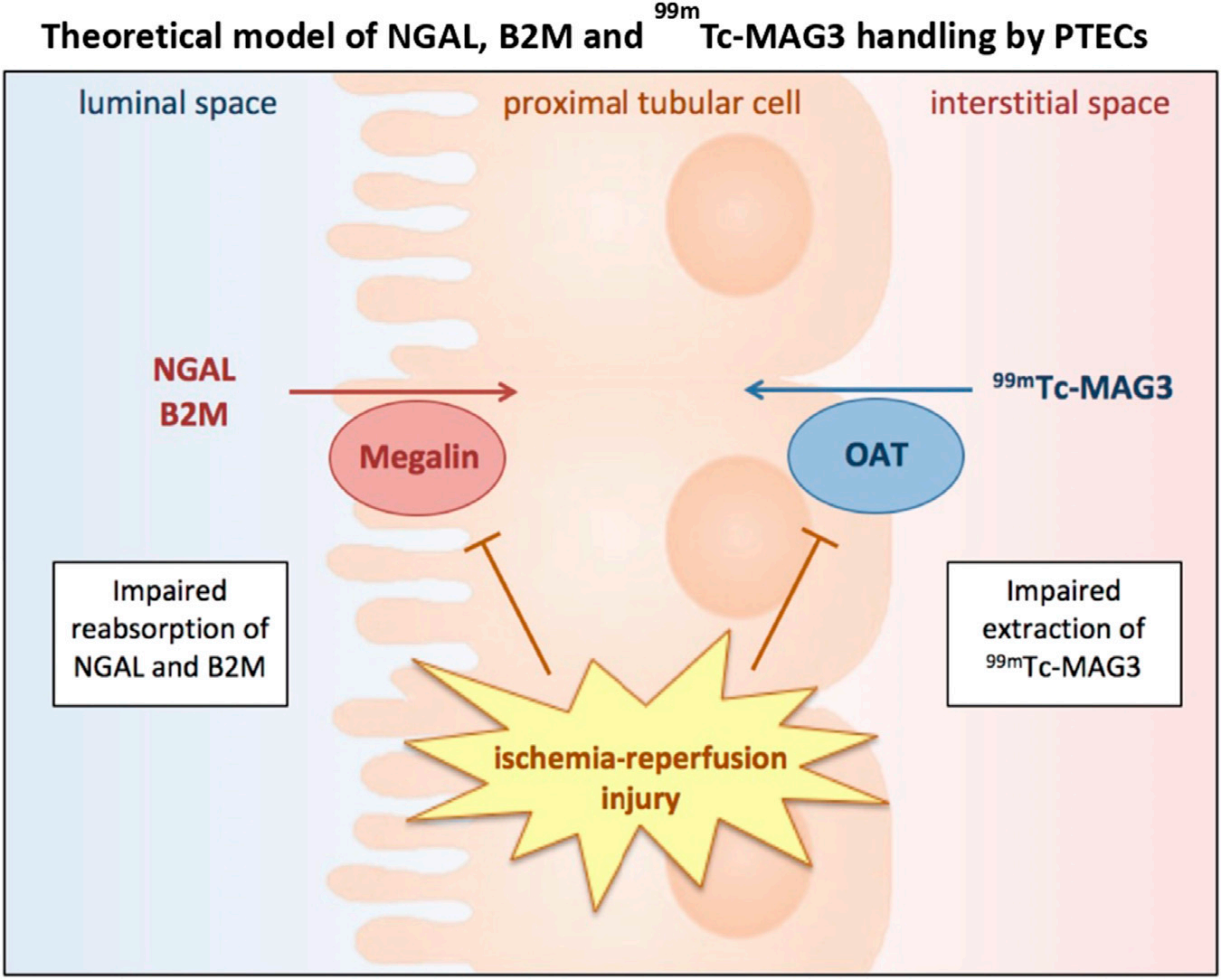
Ischemic and/or reperfusion injury of PTECs resulting in impaired ^99m^Tc-MAG3 extraction via OATs on basolateral membranes as well as impaired NGAL and B2M reabsorption via megalin on apical membranes. Abbreviations: B2M, beta-2 microglobulin; NGAL, neutrophil gelatinase-associated lipocalin; PTEC, proximal tubular epithelial cell; OAT, organic anion transporter, ^99m^Tc-MAG3, ^99m^Technetium-mercaptoacetyltriglycine.

^99m^Tc-MAG3 is transported by organic anion transporters (OAT) expressed on the basolateral side of PTECs. Urinary biomarkers that identify tubular damage may offer a safer, quicker, and cheaper alternative to ^99m^Tc-MAG3 renography. Several novel markers of urinary kidney injury, such as tissue inhibitor of metalloproteinases-2 (TIMP-2), insulin-like growth factor–binding protein 7 (IGFBP7), kidney injury molecule 1 (KIM-1), CXC Motif Chemokine Ligand 9 (CXCL9), uromodulin (UMOD), neutrophil gelatinase–associated lipocalin (NGAL) and beta-2 microglobulin (B2M) have been investigated. In particular, urinary markers of PTEC dysfunction may potentially be of interest to monitor the return of PTEC functionality. Both NGAL and B2M are freely filtered and almost completely reabsorbed via the luminal side of the PTECs (Figure 1). However, their exact pathophysiological role and diagnostic value in different etiologies of kidney injury remain unclear. Here, we investigated the relation between kidney transplant tissue quality and the duration of fDGF. Subsequently, we assessed the change in these novel urinary biomarkers of kidney injury in DCD kidney transplantation recipients stratified by fDGF duration as a measure of IRI severity. Finally, we compared the performance of these markers in predicting fDGF duration to that of TFS (chosen as the gold standard), in order to identify markers that can be used to easily monitor PTEC functionality.

## Materials and Methods

### Study Design and Population

We included 89 out of 92 DCD kidney transplant recipients who participated in the Prospective Trial on Erythropoietin in Clinical Transplantation (PROTECT) [12]. Three PROTECT participants were excluded from the current project as they experienced PNF due to early graft thrombosis and urine samples were therefore not available. Briefly, PROTECT was a randomized, double-blind study comparing high-dose recombinant human erythropoietin (EPO)-β to placebo for the combined primary endpoint of primary nonfunction and DGF. All consecutive patients scheduled to receive a DCD kidney transplant at Leiden University Medical Center between August 2005 and December 2009 were approached to participate. High-dose EPO was administered to the recipient as an intravenous bolus on 3 consecutive days (total dose 100.000 IE) starting 3–4 h before the transplantation. All donors were controlled DCD cases (Maastricht Category III). Kidneys were allocated according to the allocation algorithm and matching criteria of both the standard Eurotransplant Kidney Allocation System (ETKAS; n = 73) and the Eurotransplant Senior Program (ESP; n = 16). At that time, Super Rapid (SR) procurement with cold preservation perfusion or Normothermic Regional Perfusion (NRP) technique was not yet available. There was no donor age limit for acceptance of DCD kidneys. The median age (46 years) of the DCD cases in the Netherlands was previously found to be significantly lower than that of heart-beating donors (48.5 years) in the period before the PROTECT study commenced [13]. All consecutive patients scheduled to receive a DCD kidney transplant were approached to participate in the PROTECT study. Exclusion criteria included panel-reactive antibodies (PRAs) > 60% at the time of transplantation, donor serum creatinine >150 μmol/L, first warm ischemic time (WIT) ≥ 45 min or cold ischemic time (CIT) > 24 h. The immunosuppressive regimen consisted of induction therapy with anti-CD25 antibody (daclizumab; intravenous bolus 100 mg preoperatively and on postoperative day (POD) 10) and triple maintenance therapy with mycophenolate mofetil, corticosteroids and delayed introduction of cyclosporine A (CsA) microemulsion. CsA (initial dose 3 mg/kg twice daily) was introduced on POD 4, with subsequent dosing according to the 12-hour area under the curve (AUC) targets of 5,400 ng/mL/h for the first 6 weeks after transplantation and 3,250 ng/mL/h thereafter. All patients received prophylactic therapy with trimethoprim/sulfamethoxazole 480 mg/day for 6 months against pneumocystis jiroveci pneumonia. The study was approved by the Ethical Committee of the Leiden University Medical Center (NCT00157300). At 1 year, there was no difference in the incidence or duration of DGF and/or primary nonfunction in patients treated with high-dose EPO versus placebo. Further details and results have been described previously [12].

### Data Collection

Collected information included recipient age, sex, primary kidney disease, previous kidney transplantations, time on dialysis, donor age, sex, cause of death, hypertension, serum creatinine, and transplant characteristics including HLA mismatch, PRA, CIT and WIT. Additionally the kidney donor risk index (KDRI), a widely used tool to predict the risk of graft failure based on deceased donor characteristics, was calculated [14].

### Definition of Functional DGF (fDGF)

DGF was defined according to the functional definition (fDGF); a failure of serum creatinine to decrease spontaneously by ≥ 10% daily on three successive days during the week after transplantation or dialysis requirement [8]. The second of three consecutive days was used as the index day to group patients by duration of fDGF, resulting in 4 groups of <7 days, ≥7–14 days, ≥14–21 days and ≥21 days. fDGF ≥7 or ≥21 days can be considered as having either fDGF or severe fDGF, respectively.

### Laboratory Measures

At the time of the study, urine and serum samples were collected at PODs 1-7, POD 10, at 6 weeks and 6 months after transplantation. For the current project, we only used urine data on PODs 1, 4 and 10, at 6 weeks and 6 months after transplantation since TFS and serum analyses for creatinine, B2M and NGAL were performed only on those days. Urine osmolality was determined by freezing point depression using an Osmo-Station (Auto & Stat model OM-6060, Arkray Inc., Kyoto, Japan). Urinary total protein (TP) was determined by turbidimetry (Cat. No. 05171954190), B2M by immunoturbidimetry (Cat. No. 08047430190), and creatinine by an enzymatic method (Cat. No. 3263991190), all using a Cobas C8000 c702 (Roche Diagnostics, Mannheim, Germany).

Urinary NGAL, IGFBP7, KIM-1, TIMP2, CXCL9, UMOD, SLC22A2 and nephrin were quantified in 36 batches between January 2021 and November 2021, using an in-house developed multiplex liquid chromatography tandem mass spectrometry (LC-MS/MS) test. The preanalytical and analytical phases of this LC-MS/MS test followed the standard operating procedure described elsewhere [15, 16]. To ensure LC-MS/MS performance, a system suitability test was carried out prior to each analysis batch of study samples (a maximum of 81 samples per batch). To monitor LC-MS/MS performance over time, two urine-based internal quality control (IQC) samples were prepared and analyzed with the study samples. The IQC results were monitored in Levey-Jennings charts and the test performance was considered stable over 1 year [17, 18]. All urine samples were stored for 10–15 years and underwent one to two freeze-thaw cycles. However, it is important to note that LC-MS/MS tests are relatively insensitive to freezing and thawing of samples.

With the serum and urinary biomarker and creatinine values, fractional excretion (FE) of B2M and NGAL were calculated, analogous to the FE of sodium. In analogy to the protein-to-creatinine ratio (PCR), we calculated the ratios of B2M/(TIMP2) and NGAL/(TIMP2). Theoretically, TIMP2 or IGFBP7 could substitute creatinine as a glomerular filtration marker, whereas B2M and NGAL are actively reabsorbed in proximal epithelial cells.

### Protocol Kidney Biopsy

Per the protocol, all recipients underwent a kidney transplant biopsy on POD 10. Adequate biopsy samples were available for 64 recipients. Biopsies were unavailable (n = 25) due to: withdrawal of consent (n = 7), insufficient tissue (n = 11) or staining issues (n = 7). An experienced pathologist scored all biopsies according to the semi-quantitative ATN score and assessed interstitial fibrosis and tubular atrophy (IF/TA) according to the Banff 2009 classification, as a proxy for donor-derived fibrosis [19, 20]. Biopsy results have been published previously [21].

### ^99m^Tc-MAG3 Renography

^99m^Tc-MAG3 renography was performed on PODs 1, 4 and 10 to calculate the tubular function slope (TFS) [10]. Briefly, a bolus of 100 MBq of ^99m^Tc-MAG3 was injected and frames were recorded with a large-field-of-view gamma camera (Toshiba GCA501S), at 1-second intervals for 120 frames, then at 20-second intervals for 90 frames. The ^99m^Tc-MAG3 dose was corrected for extravasation. TFS was calculated by analyzing radiopharmaceutical uptake by renal tubular cells using a nuclear medicine computer (MAPS 10000 Web Link Medical). Two regions of interest were drawn semi-automatically; one around the graft and one representing the background. Subsequently, a background-subtracted graft and dose-adjusted ^99m^Tc-MAG3 curve were generated. During the first two minutes of the renography two phases can be recognized in the graft: a rapidly ascending phase, representing the perfusion of the kidney, followed by a second phase of tubular extraction. Using a linear fit (least-squares error estimate), the slope of the second phase of this curve was determined and defined as TFS.

### Statistical Analysis

First, baseline recipient, donor and transplant characteristics are presented here as mean (±SD) or number (proportion) for all recipients and stratified by fDGF duration. Second, we studied the relation between baseline KDRI, fDGF duration and IF/TA presence in the kidney biopsy on POD 10, using logistic regression analysis to investigate the clinical relevance of severe fDGF. Third, we studied the relation between fDGF duration and endogenous creatinine clearance (ECC) at 6 weeks and 12 months, using logistic regression analysis for the outcome ECC ≥40 vs*.* < 40 mL/min, and linear regression analysis for the change in ECC in mL/min.

Fourth, we calculated the median (interquartile range [IQR]) values of TFS, urine volume, creatinine, PCR, and creatinine-corrected NGAL, B2M, TIMP2, IGFBP7, KIM-1, CXCL9, UMOD, and FE-NGAL and FE-B2M on PODs 1, 4 and 10, for all recipients and stratified by fDGF duration.

Fifth, we calculated Pearson correlation coefficients to study the association between TFS and urinary creatinine, PCR, standardized creatinine-corrected NGAL, B2M and TIMP2, IGFBP7, KIM-1, CXCL9, UMOD, and FE-NGAL and FE-B2M, at PODs 1, 4 and 10. A *p*-value < 0.05 was considered statistically significant.

Sixth, we calculated AUCs with receiver operating characteristic (ROC) analyses for fDGF presence at PODs 1 and 4, and fDGF severity at POD 10 as predicted by standardized TFS, urinary creatinine, PCR and standardized creatinine-corrected urinary NGAL, B2M, TIMP2, IGFBP7, KIM-1, CXCL9, UMOD, FE-NGAL and FE-B2M. In all regression and ROC analyses, markers were divided by their SD to normalize their distributions.

Missing urine samples and measurements are reported in Supplementary Table S3. At PODs 4 and 10, 32 DCD kidney transplant recipients had complete data for ROC analyses. We conducted a complete case analysis to compare results with our main analyses. All analyses were performed using R version 4.0.3 (R Core Team, Vienna, Austria).

## Results

### Cohort Characteristics

Urine and serum samples were available for 89 DCD recipients. The mean age of the recipients was 54 (±13) years and 62 (70%) were men. For 86 (96%) recipients, this was their first kidney transplant, with a mean dialysis vintage of 4.4 (±2.5) years. Only one recipient (1%) received a preemptive transplant. Of the 89 recipients, 20 (22%) had no fDGF (<7 days), 20 (22%) had mild fDGF (≥7–14 days), 26 (29%) had moderate fDGF (≥14 to <21 days), and 23 (26%) had severe fDGF (≥21 days). The mean age of donors was 46 (±15) years and 55% were men. Donors for recipients with fDGF (≥7 days) were older, more often men and had higher KDRI scores than those without fDGF. At POD 10, IF/TA was more often present, TFS and ECC were lower in those with fDGF, especially severe fDGF, compared to those without fDGF. Further donor, recipient and transplant characteristics are summarized in Table 1. Detailed causes of primary kidney disease are shown in Supplementary Table S1.

**TABLE 1:** Recipient, donor, and transplant characteristics of 89 donation after circulatory death kidney transplantations.

Characteristic	All	Functional delayed graft function^a^			
		No	Mild	Moderate	Severe
*n*	89	20	20	26	23
Recipients					
Age, years	54 (±13)	50 (±13)	53 (±12)	52 (±13)	59 (±12)
Male patients, *n* (%)	62 (70)	16 (80)	13 (65)	18 (69)	15 (65)
Primary kidney disease, *n* (%)					
Diabetes, hypertension or nephrosclerosis	29 (32)	6 (30)	7 (35)	8 (31)	8 (35)
Primary or systemic glomerular disease	28 (32)	7 (35)	3 (15)	11 (42)	7 (30)
Polycystic kidney disease	16 (18)	2 (10)	6 (30)	4 (15)	4 (17)
Other or unknown	16 (18)	5 (25)	4 (20)	3 (12)	4 (17)
PRA >5%, %	9 (10)	4 (20)	0 (0)	3 (12)	2 (9)
Repeat transplant, *n* (%)	3 (3)	0 (0)	2 (10)	1 (4)	0 (0)
Pre-emptive transplant, *n* (%)	1 (1)	1 (5)	0 (0)	0 (0)	0 (0)
Dialysis vintage, y	4.4 (±2.5)	4.9 (±4.3)	3.8 (±1.7)	4.5 (±1.9)	4.4 (±1.5)
Donor					
Age, years	46 (±15)	35 (±13)	48 (±13)	48 (±13)	52 (±18)
Male, *n* (%)	49 (55)	10 (50)	8 (40)	13 (50)	18 (78)
Cause of death: CVA, *n* (%)	36 (40)	6 (30)	6 (30)	12 (46)	12 (52)
Hypertension, *n* (%)	18 (20)	1 (5)	5 (25)	6 (23)	6 (26)
Serum creatinine, μmol/L	79 (±51)	76 (±27)	74 (±29)	93 (±85)	71 (±21)
Transplant					
KDRI score, *n* (%)					
<1	34 (38)	14 (70)	7 (35)	9 (35)	4 (17)
≥1 to 1.5	36 (40)	5 (25)	10 (50)	11 (42)	10 (44)
≥1.5 to 2	15 (17)	1 (5)	2 (10)	5 (19)	7 (30)
≥2	4 (5)	0 (0)	1 (5)	1 (4)	2 (9)
CIT, h	17 (±4)	16 (±4)	16 (±4)	17 (±4)	18 (±4)
WIT I, min	18 (±6)	16 (±5)	16 (±5)	19 (±6)	20 (±7)
WIT II, min	30 (±7)	32 (±7)	30 (±8)	28 (±6)	31 (±8)
Post-transplant day 10					
IF/TA present, *n* (%)	26 (33)	2 (13)	5 (26)	4 (17)	15 (68)
TFS	1.5 (±1.1)	2.5 (±1.2)	1.9 (±1.0)	1.0 (±0.8)	0.9 (±0.8)
Serum creatinine, μmol/L	520 (±273)	195 (±90)	416 (±152)	750 (±225)	632 (±182)
Endogenous creatinine clearance, mL/min	16 (±23)	50 (±16)	22 (±16)	8 (±5)	4 (±5)
24-h urine volume, L	1.6 (±1.3)	2.7 (±0.8)	2.4 (±1.4)	1.1 (±0.8)	0.7 (±0.8)
Proteinuria, g/24u	0.8 (±1.5)	0.7 (±0.5)	0.8 (±0.4)	0.7 (±0.4)	0.5 (±0.4)

Continuous variables are expressed as mean (± standard deviation).

^a^ Defined on the basis of fDGF duration as <7, ≥7 to <14, ≥14 to <21, and≥21 for no, mild, moderate, and severe fDGF, respectively.

Abbreviations: CIT, cold ischemia time; CsA, cyclosporine A; CVA, cerebrovascular accident; DCD, donation after circulatory death; DGF, delayed graft function; fDGF, functional delayed graft function; KDRI, kidney donor risk index; PRA, panel reactive antibody; TFS, tubular function slope; WIT I, first warm ischemia time (time between clamping the aorta of the donor and cooling of the organ to 4°C); WIT II, second warm ischemia time (time during construction of vascular anastomoses and gradual heating of the organ, until removal of the aortic clamp and revascularization).

### fDGF Duration: KDRI, Donor-Derived Fibrosis and 1-Year Renal Function

Using a KDRI of <1.0 as reference, scores of ≥1.0 to 1.5 and ≥1.5 were associated with an OR (95% CI) for POD 10 kidney biopsy IF/TA presence of 8.3 (1.7; 40.9) and 15.6 (2.8; 86.8), respectively. Compared to no fDGF, fDGF was associated with an OR of 4.2 (0.9; 20.1) for IF/TA presence at POD 10. For mild and moderate compared to no fDGF, the OR for IF/TA presence was 1.9 (0.4; 10.0), whereas this was 15.0 (2.7; 84.8) for severe fDGF (Table 2).

**TABLE 2:** Risk of IF/TA according to KDRI or fDGF scores compared to the reference category in 89 DCD recipients.

Risk factor	IF/TA present at POD 10
	OR (95% CI)
KDRI score	
<1.0 (reference)	1
≥1.0 to 1.5	8.3 (1.7–40.9)
≥1.5	15.6 (2.8–86.8)
fDGF	
No (reference)	1
Yes	4.2 (0.9–20.1)
Mild and moderate	1.9 (0.4–10.0)
Severe	15.0 (2.7–84.8)

Abbreviations: DCD, donation after circulatory death; fDGF, functional delayed graft function; IF/TA, interstitial fibrosis/tubular atrophy; KDRI, kidney donor risk index; OR, odds ratio; POD, postoperative day; 95% CI, 95% confidence interval.

Longer duration of fDGF was clearly associated with a lower eGFR or ECC. The OR (95% CI) for ECC <40 vs*.* ≥ 40 mL/min at 6 weeks and 12 months for mild, moderate fDGF or severe fDGF compared to no fDGF were 5.2 (1.4; 19.3), 2.0 (0.5; 8.1) and 113.3 (10.8; 1,192.8), and 4.9 (0.6; 40.4), 3.5 (0.4; 30.5), and 9.5 (1.0; 89.0), respectively. For mild, moderate or severe compared to no fDGF, the RR (95% CI) for decrease in ECC in mL/min was −13.7 (−22.8; −4.7), −6.2 (−14.1; 1.8) at 6 weeks, and −30.4 (−39.6; −21.1), and −12.0 (−23.9; −0.1), −7.1 (−19.1; 4.9), and −24.2 (−38.7;−9.7) at 12 months, respectively (Table 3).

**TABLE 3:** Among 89 recipients, the odds and risk ratios (95% CI) for the relation between duration of fDGF and endogenous creatinine clearance at 6 weeks and 12 months, calculated using logistic and linear regression analysis.

fDGF	OR (95% CI)
	ECC <40 vs*.* ≥ 40 mL/min at week 6
No (*reference*)	1
Yes	5 (1–19)
Mild and moderate	2 (1–8)
Severe	113 (11–1,193)
	ECC <40 vs*.* ≥ 40 mL/min at month 12
No (*reference*)	1
Yes	5 (1–40)
Mild and moderate	4 (0–31)
Severe	10 (1–90)

	RR (95% CI)
	ECC in mL/min at week 6
No (*reference*)	1
Yes	−14 (−24 to −5)
Mild and moderate	−6 (−14 to 2)
Severe	−30 (−40 to −21)
	ECC in mL/min at month 12
fDGF	
No (*reference*)	1
Yes	−12 (−24 to −0)
Mild and moderate	−7 (−19 to 5)
Severe	−24 (−39 to −10)

Abbreviations: ECC, endogenous creatinine clearance; fDGF, functional delayed graft function; OR, odds ratio; RR, risk ratio; 95% CI, 95% confidence interval.

### Kidney Injury Markers, Conventional Markers and TFS Over Time

**FIGURE 2:**
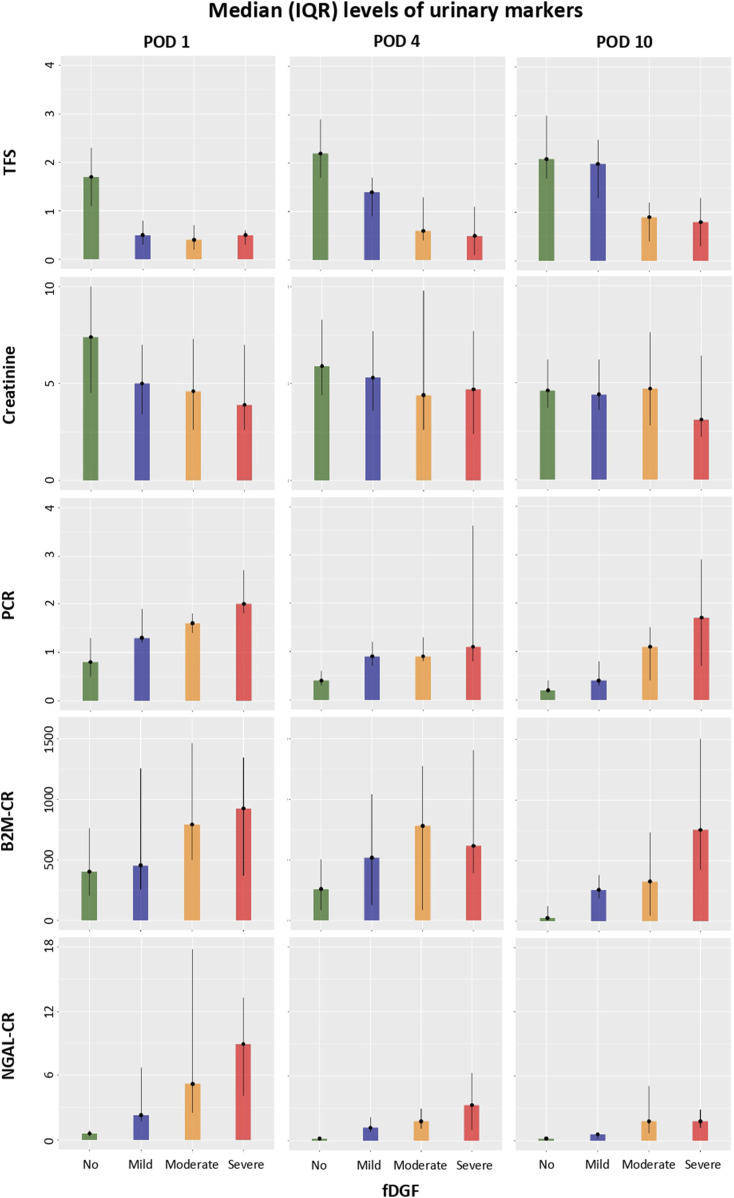
Among 89 recipients, and stratified by fDGF duration at PODs 1, 4 and 10 after DCD kidney transplantation, the median (IQR) levels of TFS and creatinine-corrected urinary markers. *Defined based on fDGF duration as <7, ≥7 to <14, ≥14 to <21, ≥21 for no, mild, moderate and severe fDGF, respectively. Abbreviations: B2M, beta-2 microglobulin; CR, creatinine ratio; DCD, donation after circulatory death; fDGF, functional delayed graft function; NGAL, neutrophil gelatinase-associated lipocalin; PCR, protein to creatinine ratio; POD, postoperative day; TFS, tubular function slope; u, urinary.

Figure 2 shows median (IQR) levels at PODs 1, 4 and 10 of TFS, conventional markers (urinary creatinine and PCR), and the urinary creatinine-standardized kidney injury markers B2M and NGAL across all recipients and stratified by fDGF duration. Detailed levels of these markers along with urine volume, TIMP2, IGFBP7, KIM1, CXCL9, UMOD and FE-B2M and FE-NGAL are shown in Supplementary Table S2 and Supplementary Figure S1. Pearson’s correlation coefficients between standardized TFS and creatinine-corrected urinary markers at PODs 1, 4 and 10 are shown in Supplementary Table S3 and Supplementary Figure S2. Correlations with TFS were generally poor or modest (as expected), with the strongest at POD 10 for urinary B2M and FE-B2M with coefficients of −0.53 (p = 0.00) and −0.56 (p = 0.00), respectively.

### Kidney Injury Markers Compared to TFS for Prediction of fDGF

**FIGURE 3:**
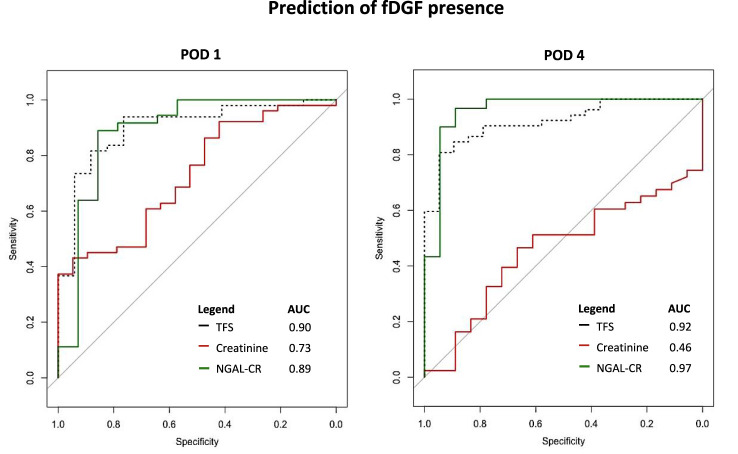
ROC analysis for the prediction of fDGF presence (yes vs*.* no) by standardized TFS and standardized creatinine-corrected urinary NGAL at PODs 1 and 4 after DCD kidney transplantation. Abbreviations: AUC, area under the curve; CR, creatinine ratio; DCD, donation after circulatory death; fDGF, functional delayed graft function; NGAL, neutrophil gelatinase-associated lipocalin; POD, postoperative day; TFS, tubular function slope.

At POD 1, AUCs (95% CI) for predicting the presence of fDGF (yes vs*.* no) were 0.90 (0.81; 0.99), 0.73 (0.60; 0.86), 0.77 (0.62; 0.92), 0.68 (0.53; 0.82), 0.55 (0.40; 0.71), 0.89 (0.76; 1.00), and 0.81 (0.68; 0.93), for TFS, creatinine, PCR, B2M, FE-B2M, NGAL and FE-NGAL, respectively. At POD 4, NGAL and FE-NGAL outperformed TFS with AUCs of 0.97 (0.90; 1.00) and 0.98 (0.93; 1.00), respectively, compared to 0.92 (0.86; 0.98) for TFS (Figure 3; Table 4; Supplementary Figure S3). At POD 10, FE-NGAL, PCR and NGAL/TIMP2 performed best for severe vs*.* mild to moderate fDGF prediction, as AUCs were 0.74 (0.55; 0.93), 0.76 (0.58; 0.94) and 0.72 (0.53; 0.91), respectively, compared to 0.65 (0.48; 0.82) for TFS (Table 4; Supplementary Figure S3). A complete case analysis including 32 DCD kidney transplant recipients yielded similar results (Supplementary Tables S4, S5).

**TABLE 4:** Area under the curve (95% CI) from ROC analysis for the prediction of fDGF presence at PODs 1 and 4, and fDGF severity at POD 10 by standardized TFS and standardized creatinine-corrected urinary markers at PODs 1, 4 and 10 after DCD kidney transplantation.

	AUC (95% CI)		
Urinary marker divided by SD	fDGF		Severe fDGF
	POD 1	POD 4	POD 10
TFS	0.90 (0.81–0.99)	0.92 (0.86–0.98)	0.65 (0.48–0.82)
Creatinine	0.73 (0.60–0.86)	0.46 (0.31–0.61)	0.65 (0.43–0.89)
P-CR	0.77 (0.62–0.92)	0.79 (0.65–0.94)	0.76 (0.58–0.94)
NGAL-CR	0.89 (0.76–1.00)	0.97 (0.90–1.00)	0.65 (0.30–1.00)
FE-NGAL	0.81 (0.68–0.93)	0.98 (0.93–1.00)	0.74 (0.55–0.93)
B2M-CR	0.68 (0.53–0.82)	0.67 (0.53–0.80)	0.69 (0.44–0.95)
FE-B2M	0.55 (0.40–0.71)	0.64 (0.50–0.78)	0.68 (0.43–0.94)
TIMP2-CR	0.87 (0.76–0.98)	0.88 (0.79–0.97)	0.69 (0.31–1.00)
IGFBP7-CR	0.81 (0.69–0.94)	0.77 (0.63–0.91)	0.52 (0.13–0.91)
KIM1-CR	0.51 (0.34–0.69)	0.45 (0.29–0.61)	0.64 (0.18–1.00)
CXCL9-CR	0.78 (0.62–0.94)	0.72 (0.53–0.91)	0.65 (0.32–0.97)
UMOD-CR	0.78 (0.66–0.90)	0.62 (0.47–0.78)	0.73 (0.38–1.00)

Abbreviations: AUC, area under the curve; B2M, beta-2 microglobulin; CR, creatinine ratio; DCD, donation after circulatory death; ECC, endogenous creatinine clearance; fDGF, functional delayed graft function; FE, fractional excretion; NGAL, neutrophil gelatinase-associated lipocalin; P-CR, protein to creatinine ratio; POD, postoperative day; TIMP2, tissue inhibitor of metalloproteinases-2; TFS, tubular function slope.

## Discussion

NGAL and FE-NGAL, measured at PODs 1, 4 and 10 outperformed TFS in predicting fDGF presence and severity in 89 DCD kidney transplantation recipients. fDGF severity was strongly related to IF/TA presence in POD 10 kidney biopsies and lower kidney function at 6 weeks and 12 months after transplantation. Daily monitoring of urinary NGAL or FE-NGAL in the first days after kidney transplantation may provide an alternative to sequential ^99m^TcMAG3 renography to follow PTEC function recovery and fDGF resolution.

DGF is associated with acute rejection and has an adverse impact on longer-term kidney function and patient outcomes [8, 22, 23]. Indeed, in our study the severity of fDGF was strongly associated with the presence of (donor-derived) IF/TA in the kidney biopsies, observed in 68% of those with fDGF ≥21 days compared to only 33% for the entire cohort. Distinguishing early acute rejection from a primarily insufficient renal mass remains challenging in the first weeks after kidney transplantation [9]. This is especially true in DCD kidney recipients in whom severe IRI is higher than in those receiving kidneys from living or DBD donors [1, 2]. We used the TFS as the gold standard to assess the severity of kidney injury. The TFS quantifies the tubular extraction rate of ^99m^Tc-MAG3 through the OAT, providing insight into the overall quality and functional recovery of PTECs. Although proven accurate and effective in identifying DGF, ^99m^Tc-MAG3 renography remains invasive, time-consuming and costly, rendering sequential TFS less appealing for routine clinical use. Our results indicate that, among both conventional and novel urinary biomarkers, FE-NGAL in particular has the potential to replace TFS allowing daily monitoring of IRI resolution.

The urinary biomarkers TIMP-2, IGFBP7, KIM-1, CXCL9, UMOD, NGAL and B2M may reflect different aspects of renal pathophysiology, although research is ongoing. For example, TIMP-2 and IGFBP7 are thought to act as markers of cellular stress and G1 cell cycle arrest, aiding in the early detection of AKI [24–26]. KIM-1 is a marker of kidney injury, primarily expressed in damaged proximal tubular cells [24, 27, 28]. CXCL9 is associated with the immune response and renal inflammation. UMOD, on the other hand, is the most abundant protein in normal urine and plays a role in kidney function and urinary tract maintenance [24].

Urinary B2M and NGAL have been well-researched as markers of proximal tubular dysfunction. B2M binds to major histocompatibility complex I (MHC-I)/human leukocyte antigen I (HLA-I) on nucleated cells [29]. NGAL is synthesized by epithelial tissues, including distal tubular epithelial cells [29]. In normal kidneys, B2M and NGAL undergo unhindered glomerular filtration and are almost entirely reabsorbed by PTECs [27, 29, 30]. After any surgical procedure that causes damage to the epithelial tissue, both plasma B2M and NGAL will increase. Following PTEC injury, reabsorption of B2M and NGAL is impaired, increasing urinary excretion. Since B2M and NGAL are normally reabsorbed via the apical PTEC membrane, and ^99m^Tc-MAG_3_ (used to determine the TFS) at the basolateral membrane, we hypothesized that these markers specifically would be equally accurate in the prediction of fDGF. However, since B2M (as compared to NGAL) is more abundantly present in tissues, blood levels of B2M will increase more than NGAL following surgery. The subsequent high fractional urinary excretion of B2M, independent of PTEC function, disrupts the interpretability of urinary B2M as a marker of PTEC injury. Indeed, we found that compared to TFS, NGAL-CR or FE-NGAL were stronger predictors than B2M-CR for the presence of fDGF at POD 1 and POD 4, and severe fDGF at POD 10. Thus, NGAL-CR and FE-NGAL may provide an alternative to ^99m^TcMAG3 renography for following fDGF resolution and anticipating poorer long-term kidney function.

**FIGURE 4:**
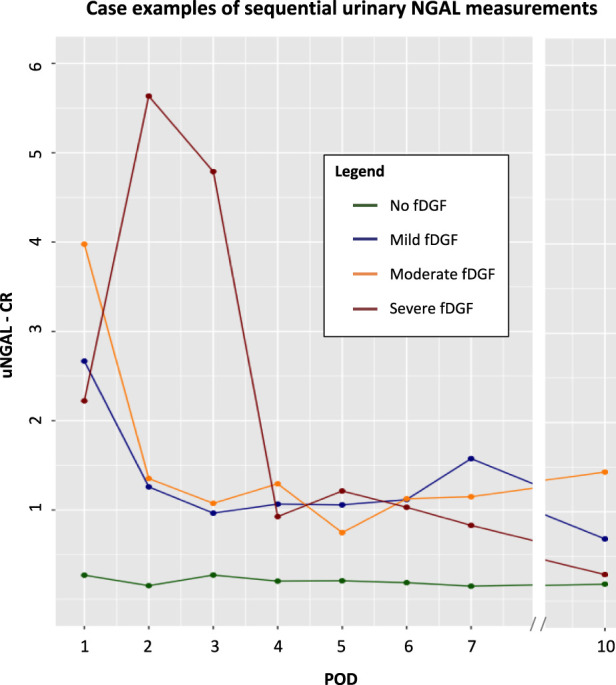
Case examples of urinary (creatinine-corrected) NGAL measurements among 4 representative DCD kidney transplantation recipients with no, mild, moderate or severe fDGF*. *Defined based on fDGF duration as <7, ≥7 to <14, ≥14 to <21, ≥21 days for no, mild, moderate and severe fDGF, respectively. Abbreviations: CR, creatinine ratio; DCD, donation after circulatory death; fDGF, functional delayed graft function; NGAL, neutrophil gelatinase-associated lipocalin; POD, postoperative day; u, urinary.

Sequential measurements of NGAL-CR or FE-NGAL in the first days following kidney transplantation can be used to monitor the recovery of PTEC function. Through this, transplant recipients prone to experience severe fDGF may be identified early on, as NGAL will not yet be reabsorbed due to PTEC dysfunction. A decrease in urinary NGAL will indicate restoration of PTEC functionality (Figure 4). In our DCD transplant recipient population, (FE-)NGAL levels (corrected for urinary creatinine and the population standard deviation) on separate days already predicted the presence and severity of fDGF when compared to the TFS as the gold standard. Sequential monitoring of NGAL will be more informative, especially considering the fluctuating nature and wide interpatient variation of NGAL. Of course, future research in larger populations is needed to determine reference levels and to interpret when a relative increase or decrease in NGAL would be clinically relevant. Furthermore, NGAL levels should always be interpreted in the context of other clinical characteristics such as diuretic volume. Decreasing urinary NGAL levels, indicating recovery of PTEC function, could guide the indication for or timing of kidney transplant biopsy to exclude the occurrence of another acute rejection episode and subsequent treatment. Therefore, in the future, NGAL testing may also improve long-term outcomes.

The current body of evidence is, however, insufficient to support large-scale implementation of routine NGAL measurement. Furthermore, sequential NGAL measurement could also be beneficial for other indications other than DGF resolution monitoring, such as early AKI monitoring, for instance during treatment with potentially nephrotoxic chemotherapeutics. If sequential NGAL testing is validated in future research as reliable for monitoring DGF resolution or timely diagnosis of early AKI, it could guide timely intervention and thereby significantly reduce costs by preventing severe complications [31, 32]. Finally, with the development of easier analytical techniques, such as our in-house developed multiplex liquid chromatography tandem mass spectrometry (LC-MS/MS) test, biomarker analysis costs will be reduced [15, 16].

There are several strengths to our study. First, we are comparing urinary markers to the TFS (^99m^TcMAG3 renography) shortly after DCD kidney transplantation for the first time. Since recipients of DCD kidneys are at higher risk for severe IRI, adequate monitoring of kidney injury is especially important in this group. Second, by using an in-house developed multiplex LC-MS/MS test, we were able to efficiently and reliably assess urinary levels of nine injury markers simultaneously. Third, by comparing previously proposed markers of kidney injury in recipients of DCD kidneys at high risk of severe IRI, we aimed to perform a hypothesis-generating study that may focus future research efforts. Fourth, there was no selection of participants in the PROTECT study since all consecutive patients scheduled to receive a DCD kidney transplant were approached to participate. This included expanded criteria donors (ECD) and kidneys allocated via the Eurotransplant Senior Program (ESP). Fifth, organ procurement techniques have significantly improved since the time of the PROTECT study (2005–2009). Nowadays, procurement techniques such as SR and NRP reduce the severity of IRI in DCD kidney transplantation. Consequently, with more heterogeneity in IRI severity among current DCD kidney recipients, the application of these biomarkers (after external validation) for early fDGF prediction may become especially relevant. Our results should ideally be validated in representative cohorts that include these different options in procurement and allocation strategies.

Nevertheless, our study has some limitations. First, this was a single-center study, which may limit the generalizability of our results to other centers or countries. However, our experience with DCD kidney transplantation allowed us to use this relatively large cohort to investigate the added value and patterns of these biomarkers in DCD kidney recipients. Second, we found a high percentage of IF/TA in the kidney transplant biopsies at POD 10. This is to be expected considering that these were DCD kidney transplant recipients who were transplanted between August 2005 and December 2009. The consecutive DCD kidneys offered included expanded criteria donors (ECD) and kidneys allocated via the Eurotransplant Senior Program (ESP). The presence of IF/TA was strongly related to the KDRI score, which is largely driven by donor age. Hence, IF/TA presence will mainly be donor-derived. The high percentage of IF/TA in these DCD kidney transplants will have affected the levels of urinary biomarkers. Hence, these results should not be generalized to cohorts other than DCD kidney transplant recipients. Furthermore, as transplantation techniques have improved since 2009, our results should ideally be validated in representative cohorts that include these different options in procurement and allocation strategies. Third, no data exist on whether the high-dose EPO administered as an intervention to a part of the study population may have affected the validity of our biomarker analyses. However, as there was no difference in the incidence or duration of DGF and/or primary nonfunction in patients treated with high-dose EPO versus placebo, we do not expect EPO administration to have affected our biomarker results. Fourth, due to the limited sample size and missing data, this study may have been underpowered to some extent, particularly to detect associations between urinary biomarker levels and concurrent kidney function and kidney function decline. This will, however, not have been inferred from our descriptive analysis assessing the patterns of these urinary biomarkers and their correlation with TFS. Third, part of the missing urine samples will be due to anuria. However, since anuria itself indicates DGF, it will be less relevant to measure biomarker levels in anuric patients. Finally, all urine samples included in our analysis were stored for 10–15 years and underwent one to two freeze-thaw cycles. However, it is important to note that LC-MS/MS tests are relatively insensitive to freezing and thawing of samples.

In conclusion, we found that NGAL and FE-NGAL, measured on PODs 1, 4 or 10 after DCD kidney transplantation in 89 recipients, performed better than the TFS in predicting the presence and severity of fDGF. fDGF severity was strongly related to the KDRI, the presence of donor-derived fibrosis in day-10 protocol kidney biopsies, and resulted in inferior kidney graft function 12 months after kidney transplantation. Daily urinary NGAL and FE-NGAL monitoring in the first days after kidney transplantation may provide an alternative to sequential ^99m^TcMAG3 renography to follow PTEC function recovery and fDGF resolution, and may guide the timing of a kidney biopsy to exclude the occurrence of an additional acute rejection episode.

## Data Availability

The datasets presented in this article are not readily available because as our data could be used to identify individuals, privacy concerns prevent us from allowing them to be publically available. Nonetheless, we are open to make our data available for collaboration conditional on agreement on privacy matters and appropriate usage of the data. For this, please contact the corresponding author. Requests to access the datasets should be directed to ER, e.n.m.rooij@lumc.nl.
