# Supplementary material for: Urinary NGAL Outperforms 99mTc-MAG3 Renography in Predicting DCD Kidney Graft Function
Source: Transpl Int. 2025 May 12;38:13818. doi: 10.3389/ti.2025.13818 (PMC12104080; doi:10.3389/ti.2025.13818)
Supplement: Supplementary file 1 [file DataSheet1.DOCX]

**Supplemental material**

| **Supplemental Table 1.** Cause of primary kidney disease in detail, stratified by fDGF. | | | | | |
| --- | --- | --- | --- | --- | --- |
|  |  | **fDGF*** | | | |
| **Characteristic** | **All** | **No** | **Mild** | **Moderate** | **Severe** |
| *n* | 89 | 20 | 20 | 26 | 23 |
| Primary kidney disease, *n* (%) |  |  |  |  |  |
| *Diabetes and hypertension* |  |  |  |  |  |
| Diabetic nephropathy | 11 (12) | 0 (0) | 5 (25) | 3 (12) | 3 (13) |
| Nephrosclerosis | 15 (17) | 5 (25) | 2 (10) | 4 (15) | 4 (18) |
| Hypertensive nephropathy | 3 (3) | 1 (5) | 0 (0) | 1 (4) | 1 (4) |
| *Polycystic kidney disease* |  |  |  |  |  |
| ADPKD | 15 (17) | 2 (10) | 6 (30) | 4 (15) | 3 (13) |
| Medullary cystic kidney disease | 1 (1) | 0 (0) | 0 (0) | 0 (0) | 1 (4) |
| *Glomerulonephritis* |  |  |  |  |  |
| IgA nephropathy | 13 (15) | 3 (15) | 1 (5) | 5 (19) | 4 (18) |
| Focal segmental glomerulosclerosis | 4 (4) | 1 (5) | 1 (5) | 2 (7) | 0 (0) |
| MELAS syndrome | 1 (1) | 0 (0) | 0 (0) | 0 (0) | 1 (4) |
| Membranoproliferative glomerulonephritis | 3 (3) | 1 (5) | 0 (0) | 1 (4) | 1 (4) |
| Membranous nephropathy | 1 (1) | 0 (0) | 0 (0) | 1 (4) | 0 (0) |
| Glomerulonephritis, not further specified | 1 (1) | 0 (0) | 0 (0) | 1 (4) | 0 (0) |
| *Autoimmune diseases* |  |  |  |  |  |
| Granulomatosis with polyangiitis | 3 (3) | 1 (5) | 1 (5) | 1 (4) | 0 (0) |
| Henoch Schonlein purpura nephritis | 1 (1) | 1 (5) | 0 (0) | 0 (0) | 0 (0) |
| Anti–glomerular basement membrane disease | 1 (1) | 0 (0) | 0 (0) | 0 (0) | 1 (4) |
| *Hereditary or congenital* |  |  |  |  |  |
| Alport syndrome | 1 (1) | 1 (5) | 0 (0) | 0 (0) | 0 (0) |
| Congenital disorder | 1 (1) | 0 (0) | 1 (5) | 0 (0) | 0 (0) |
| Hereditary, not further specified | 2 (2) | 1 (5) | 0 (0) | 1 (4) | 0 (0) |
| *Postrenal causes* |  |  |  |  |  |
| Vesicoureteral reflux | 3 (3) | 2 (10) | 0 (0) | 0 (0) | 1 (4) |
| Kidney stones | 1 (1) | 0 (0) | 1 (5) | 0 (0) | 0 (0) |
| Carcinoma | 1 (1) | 1 (5) | 0 (0) | 0 (0) | 0 (0) |
| *Other* |  |  |  |  |  |
| Tubulointerstitial nephritis | 1 (1) | 0 (0) | 0 (0) | 1 (4) | 0 (0) |
| Phenacetin abuse | 1 (1) | 0 (0) | 0 (0) | 0 (0) | 1 (4) |
| Hemolytic–uremic syndrome | 1 (1) | 0 (0) | 1 (5) | 0 (0) | 0 (0) |
| *Unknown* | 4 (4) | 0 (0) | 1 (5) | 1 (4) | 2 (9) |
| *Defined based on fDGF duration as <7, ≥7 to <14, ≥14 to <21, ≥21 for no, mild, moderate and severe fDGF, respectively.  Abbreviations: fDGF, functional delayed graft function; ADPKD, autosomal dominant polycystic kidney disease; MELAS, mitochondrial encephalomyopathy, lactic acidosis and stroke-like episodes. | | | | | |

| **Supplemental Table 2.**  Among 89 recipients, and stratified by fDGF duration on POD 1, 4 and 10 after DCD kidney transplantation, the median (IQR) levels of TFS, and (creatinine-corrected) urinary markers. | | | | | | |
| --- | --- | --- | --- | --- | --- | --- |
|  |  |  | **fDGF duration (days)** | | | |
| **Predictor** | **POD** | **All** | **<7** | **≥7 and <14** | **≥14 and <21** | **≥21** |
| *n* |  | 89 | 20 | 20 | 26 | 23 |
| **TFS** |  |  |  |  |  |  |
|  | 1 | 0.5 (0.3-1.2) | 1.7 (1.1-2.3) | 0.5 (0.3-0.8) | 0.4 (0.2-0.7) | 0.5 (0.3-0.6) |
|  | 4 | 1.2 (0.5-1.9) | 2.2 (1.7-2.9) | 1.4 (0.9-1.7) | 0.6 (0.4-1.3) | 0.5 (0.1-1.1) |
|  | 10 | 1.3 (0.7-2.2) | 2.1 (1.7-3.0) | 2.0 (1.3-2.5) | 0.9 (0.4-1.2) | 0.8 (0.3-1.3) |
| **Urinary markers** | |  |  |  |  |  |
| uVolume |  |  |  |  |  |  |
| L/24h | 1 | 0.5 (0.1-1.2) | 1.9 (1.3-2.9) | 0.5 (0.3-0.8) | 0.3 (0.0-0.8) | 0.2 (0.0-0.5) |
|  | 4 | 0.9 (0.1-2.2) | 2.5 (2.1-3.5) | 0.9 (0.4-1.8) | 0.3 (0.1-1.5) | 0.3 (0.0-0.8) |
|  | 10 | 1.5 (0.6-3.3) | 2.5 (2.0-3.3) | 2.1 (1.5-3.0) | 1.0 (0.3-1.6) | 0.3 (0.1-1.0) |
| *Filtration* |  |  |  |  |  |  |
| uCreatinine |  |  |  |  |  |  |
| mmol/L | 1 | 4.9 (3.4-8.2) | 7.4 (4.5-10.0) | 5.0 (3.4-7.0) | 4.6 (2.6-7.3) | 3.9 (2.6-7.0) |
|  | 4 | 5.7 (3.2-8.1) | 5.9 (4.4-8.3) | 5.3 (3.6-7.7) | 4.4 (2.6-9.8) | 4.7 (2.4-7.7) |
|  | 10 | 4.2 (2.9-6.5) | 4.6 (3.7-6.2) | 4.4 (3.6-6.2) | 4.7 (2.8-7.6) | 3.1 (2.2-6.4) |
| uTIMP2-CR |  |  |  |  |  |  |
|  | 1 | 0.24 (0.08-1.14) | 0.07 (0.04-0.10) | 0.24 (0.11-0.55) | 0.35 (0.24-1.81) | 0.53 (0.35-3.22) |
|  | 4 | 0.10 (0.05-0.52) | 0.05 (0.03-0.06) | 0.11 (0.07-0.67) | 0.37 (0.22-0.76) | 0.54 (0.16-0.88) |
|  | 10 | 0.08 (0.05-0.20) | 0.05 (0.04-0.08) | 0.07 (0.04-0.09) | 0.23 (0.09-0.43) | 0.38 (020-0.49) |
| uIGFBP7-CR |  |  |  |  |  |  |
|  | 1 | 0.09 (0.07-0.25) | 0.06 (0.04-0.07) | 0.09 (0.07-0.16) | 0.17 (0.08-0.40) | 0.24 (0.11-0.40) |
|  | 4 | 0.10 (0.06-0.18) | 0.13 (0.08-0.23) | 0.12 (0.07-0.19) | 0.12 (0.07-0.19) | 0.14 (0.11-0.25) |
|  | 10 | 0.11 (0.08-0.15) | 0.09 (0.07-0.13) | 0.12 (0.09-0.15) | 0.13 (0.11-0.20) | 0.11 (0.08-0.13) |
| *Proximal tubulus function* | |  |  |  |  |  |
| uP-CR |  |  |  |  |  |  |
|  | 1 | 1.3 (0.6-1.8) | 0.8 (0.5-1.3) | 1.3 (1.2-1.9) | 1.6 (1.4-1.8) | 2.0 (1.8-2.7) |
|  | 4 | 0.8 (0.4-1.2) | 0.4 (0.3-0.6) | 0.9 (0.7-1.2) | 0.9 (0.8-1.3) | 1.1 (0.8-3.6) |
|  | 10 | 0.5 (0.3-1.2) | 0.2 (0.2-0.4) | 0.4 (0.3-0.8) | 1.1 (0.4-1.5) | 1.7 (0.7-2.9) |
| uB2M-CR |  |  |  |  |  |  |
|  | 1 | 567 (315-1224) | 402 (206-762) | 454 (256-1256) | 791 (498-1463) | 924 (369-1343) |
|  | 4 | 525 (111-1037) | 259 (87-504) | 517 (127-1041) | 780 (86-1272) | 615 (389-1404) |
|  | 10 | 244 (44-648) | 25 (15-125) | 259 (188-382) | 328 (44-733) | 754 (424-1506) |
| FE-B2M |  |  |  |  |  |  |
|  | 1 | 0.40 (0.19-0.68) | 0.32 (0.19-0.59) | 0.40 (0.09-0.53) | 0.41 (0.23-0.78) | 0.46 (0.21-0.67) |
|  | 4 | 0.33 (0.08-0.54) | 0.17 (0.08-0.34) | 0.40 (0.08-0.51) | 0.41 (0.06-0.68) | 0.46 (0.16-0.72) |
|  | 10 | 0.13 (0.03-0.41) | 0.02 (0.01-0.09) | 0.21 (0.11-0.34) | 0.13 (0.03-0.40) | 0.45 (0.26-0.57) |
| uNGAL-CR |  |  |  |  |  |  |
|  | 1 | 2.3 (0.9-7.7) | 0.6 (0.3-0.9) | 2.3 (1.7-6.7) | 5.2 (2.5-17.8) | 8.9 (4.1-13.3) |
|  | 4 | 0.9 (0.3-2.4) | 0.2 (0.1-0.3) | 1.2 (0.8-2.2) | 1.8 (1.1-3.0) | 3.3 (1.0-6.3) |
|  | 10 | 0.5 (0.2-1.4) | 0.2 (0.1-0.3) | 0.6 (0.2-0.7) | 1.8 (0.7-5.1) | 1.8 (1.2-2.9) |
| FE-NGAL |  |  |  |  |  |  |
|  | 1 | 31.3 (11.0-70.4) | 8.6 (4.4-19.3) | 22.5 (16.7-57.0) | 65.9 (30.3-98.4) | 42.9 (25.5-88.9) |
|  | 4 | 19.4 (4.8-50.3) | 1.2 (0.5-2.0) | 31.8 (11.8-67.3) | 31.7 (16.3-47.1) | 45.8 (33.5-59.2) |
|  | 10 | 2.7 (0.6-18.6) | 0.4 (0.2-0.9) | 2.5 (1.0-6.3) | 21.7 (2.7-34.7) | 17.8 (7.8-29.6) |
| *Distal tubulus function* | |  |  |  |  |  |
| uOsmolality |  |  |  |  |  |  |
| mOsm/kg | 1 | 311 (296-340) | 353 (308-367) | 308 (295-325) | 305 (294-322) | 313 (295-323) |
|  | 4 | 313 (287-384) | 405 (351-488) | 311 (294-324) | 300 (223-330) | 290 (228-321) |
|  | 10 | 325 (273-395) | 436 (398-462) | 357 (323-387) | 288 (256-341) | 277 (269-299) |
| uUMOD-CR |  |  |  |  |  |  |
|  | 1 | 0.00 (0.00-0.00) | 0.00 (0.00-0.00) | 0.00 (0.00-0.00) | 0.00 (0.00-0.00) | 0.00 (0.00-0.00) |
|  | 4 | 0.00 (0.00-0.00) | 0.00 (0.00-0.00) | 0.00 (0.00-0.00) | 0.00 (0.00-0.00) | 0.00 (0.00-0.00) |
|  | 10 | 0.00 (0.00-0.00) | 0.00 (0.00-0.00) | 0.00 (0.00-0.00) | 0.00 (0.00-0.00) | 0.00 (0.00-0.00) |
| *Interstitial inflammation* | |  |  |  |  |  |
| uCXCL9-CR |  |  |  |  |  |  |
|  | 1 | 0.01 (0.00-0.01) | 0.00 (0.00-0.00) | 0.01 (0.00-0.01) | 0.01 (0.00-0.02) | 0.01 (0.01-0.02) |
|  | 4 | 0.00 (0.00-0.01) | 0.00 (0.00-0.00) | 0.01 (0.00-0.02) | 0.00 (0.00-0.01) | 0.01 (0.01-0.01) |
|  | 10 | 0.00 (0.00-0.00) | 0.00 (0.00-0.00) | 0.00 (0.00-0.00) | 0.00 (0.00-0.00) | 0.00 (0.00-0.27) |
| *Regeneration* |  |  |  |  |  |  |
| uKIM1-CR |  |  |  |  |  |  |
|  | 1 | 0.01 (0.01-0.02) | 0.01 (0.01-0.02) | 0.01 (0.01-0.02) | 0.01 (0.01-0.01) | 0.01 (0.01-0.02) |
|  | 4 | 0.01 (0.01-0.03) | 0.01 (0.01-0.02) | 0.02 (0.01-0.05) | 0.01 (0.01-0.10) | 0.01 (0.01-0.07) |
|  | 10 | 0.02 (0.01-0.06) | 0.02 (0.01-0.02) | 0.03 (0.02-0.03) | 0.04 (0.02-0.07) | 0.02 (0.01-0.02) |
| B2M, beta-2 microglobulin; DCD, donation after circulatory death; ECC, endogenous creatinine clearance; FE, fractional excretion; IGFBP7, insulin-like growth factor-binding protein 7; NGAL, neutrophil gelatinase-associated lipocalin; PCR, protein to creatinine ratio; POD, postoperative day; TIMP2, tissue inhibitor of metalloproteinases-2; TFS, tubular function slope. | | | | | | |

**Median (IQR) urinary marker levels on POD 1, 4 and 10**

**fDGF***

**No fDGF**

Mild

Moderate

Severe

**All**


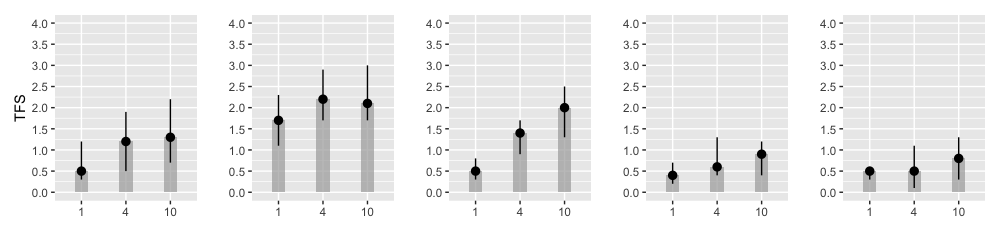


TFS


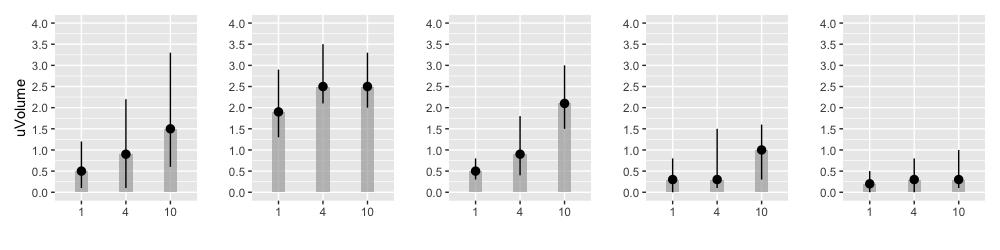


uVolume


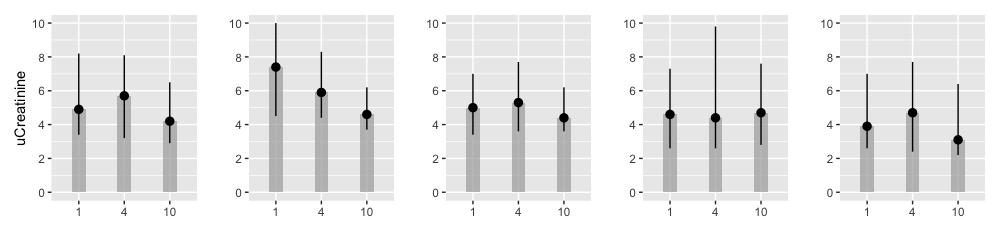


uCreatinine


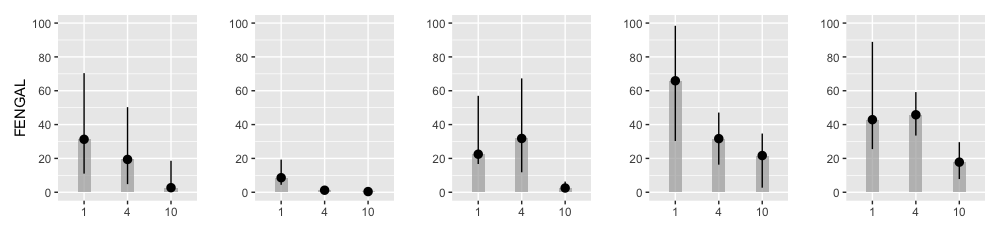

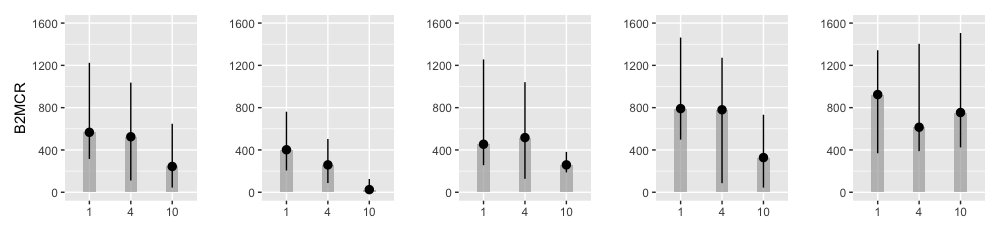

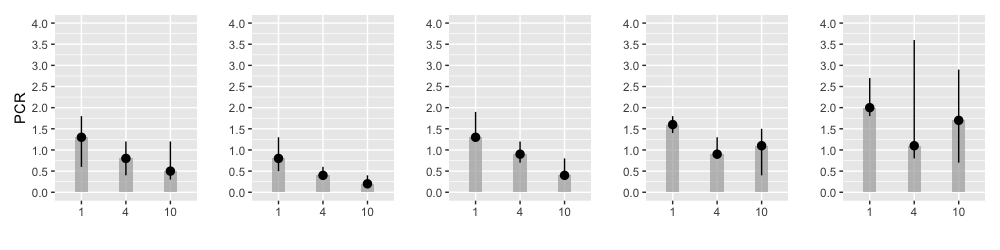

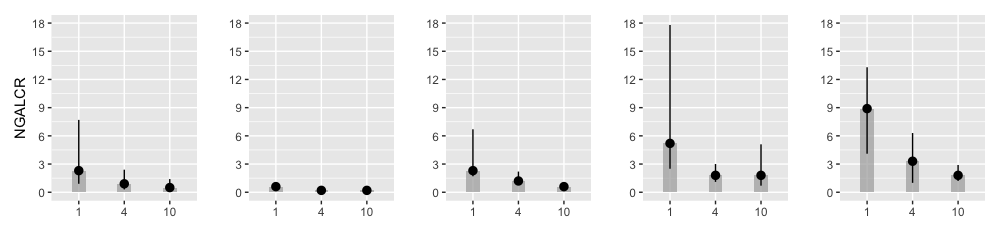


B2M-CR

FENGAL

NGAL-CR

P-CR

POD

**All**

**No fDGF**

Mild

Moderate

Severe

**fDGF***


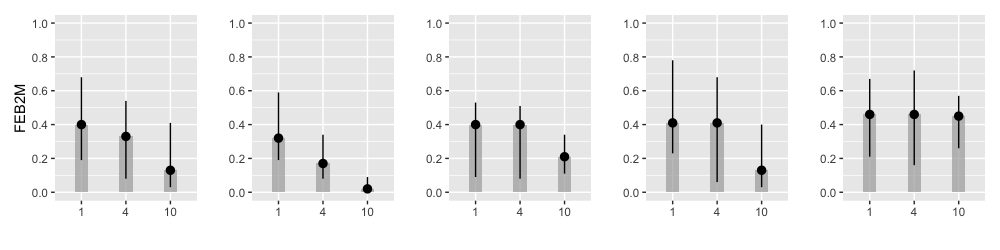


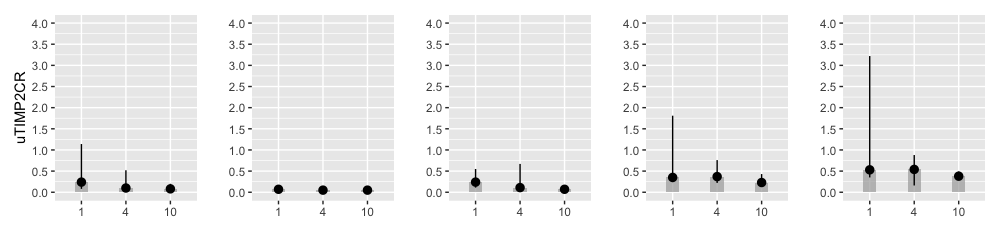

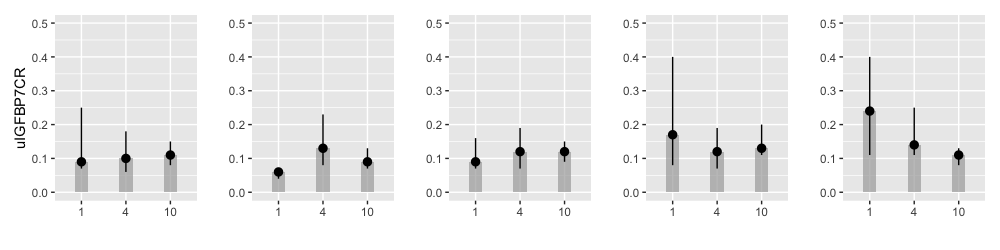


FEB2M

**Supplemental Figure 1**. Among 89 recipients, and stratified by fDGF duration at day 1, 4 and 10 after DCD kidney transplantation, the median (IQR) levels of TFS and (creatinine-corrected) urinary markers. *Defined based on fDGF duration as <7, ≥7 to <14, ≥14 to <21, ≥21 for no, mild, moderate and severe fDGF, respectively. *Abbreviations*: B2M, beta-2 microglobulin; CR, creatinine ratio; DCD, donation after circulatory death; fDGF, functional delayed graft function; FE, fractional excretion; IGFBP7, insulin-like growth factor-binding protein 7; NGAL, neutrophil gelatinase-associated lipocalin; PCR, protein to creatinine ratio; POD, postoperative day; TIMP2, tissue inhibitor of metalloproteinases-2; TFS, tubular function slope; u, urinary.

POD

IGFBP7-CR

TIMP2-CR

| **Supplemental Table 3.** Pearson correlation coefficients for the correlation between standardized TFS and standardized creatinine-corrected urinary markers on POD 1, 4 and 10 after DCD kidney transplantation. | | | |
| --- | --- | --- | --- |
|  | **Correlation coefficient for TFS, divided by SD** | | |
| **Urinary marker, divided by SD** | **POD 1** | **POD 4** | **POD 10** |
| Creatinine | 0.31 (*p* = 0.01) | -0.04 (*p* = 0.74) | 0.13 (*p* = 0.36) |
| P-CR | -0.49 (*p* = 0.00) | -0.39 (*p* = 0.01) | -0.41 (*p* = 0.00) |
| NGAL-CR | -0.28 (*p* = 0.06) | -0.17 (*p* = 0.25) | -0.22 (*p* = 0.16) |
| FE-NGAL | -0.13 (*p* = 0.30) | -0.14 (*p* = 0.29) | -0.28 (*p* = 0.04) |
| B2M-CR | -0.28 (*p* = 0.03) | -0.31 (*p* = 0.02) | -0.53 (*p* = 0.00) |
| FE-B2M | -0.15 (*p* = 0.24) | -0.36 (*p* = 0.00) | -0.56 (*p* = 0.00) |
| TIMP2-CR | -0.49 (*p* = 0.00) | -0.16 (*p* = 0.26) | -0.19 (*p* = 0.21) |
| IGFBP7-CR | -0.24 (*p* = 0.09) | -0.14 (*p* = 0.34) | -0.22 (*p* = 0.15) |
| KIM1-CR | 0.06 (*p* = 0.69) | -0.08 (*p* = 0.59) | -0.11 (*p* = 0.48) |
| CXCL9-CR | -0.25 (*p* = 0.10) | -0.26 (*p* = 0.10) | -0.24 (*p* = 0.29) |
| UMOD-CR | 0.18 (*p* = 0.19) | 0.14 (*p* = 0.33) | 0.00 (*p* = 0.95) |
| *Abbreviations*: B2M, beta-2 microglobulin; CR, creatinine ratio; DCD, donation after circulatory death; FE, fractional excretion; NGAL, neutrophil gelatinase-associated lipocalin; P-CR, protein to creatinine ratio; POD, postoperative day; SD, standard deviation; TIMP2, tissue inhibitor of metalloproteinases-2; TFS, tubular function slope. | | | |


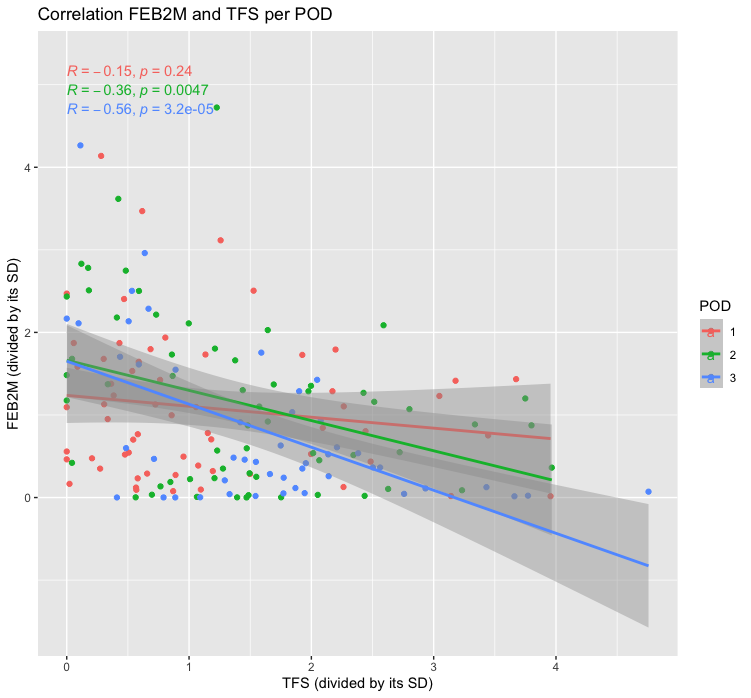

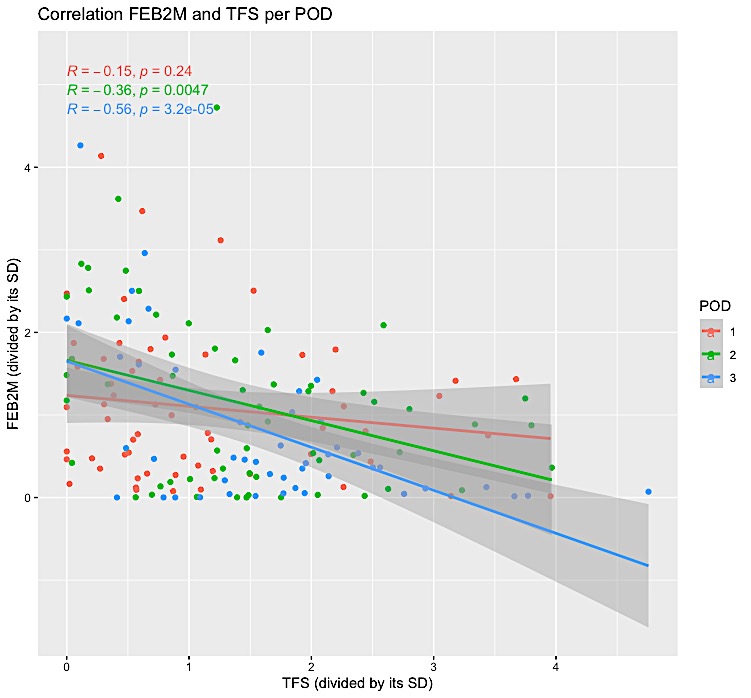

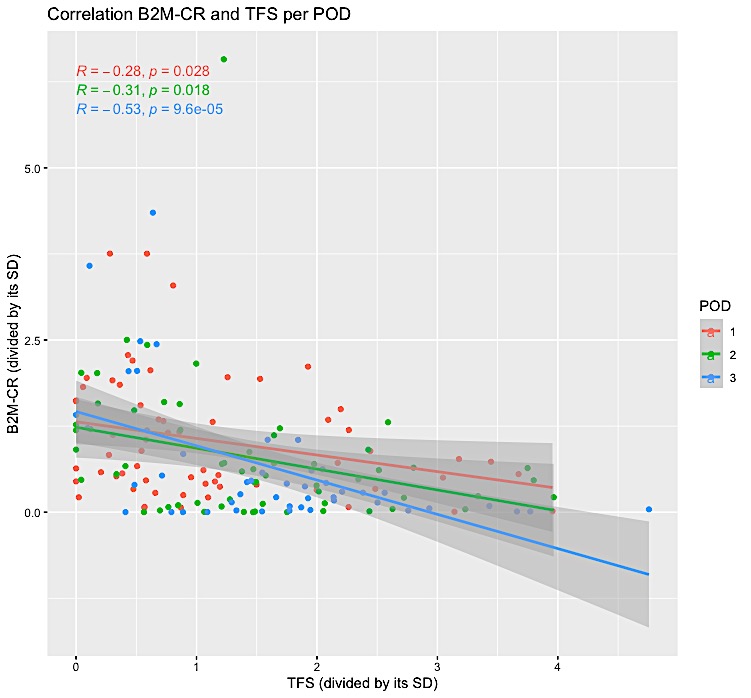

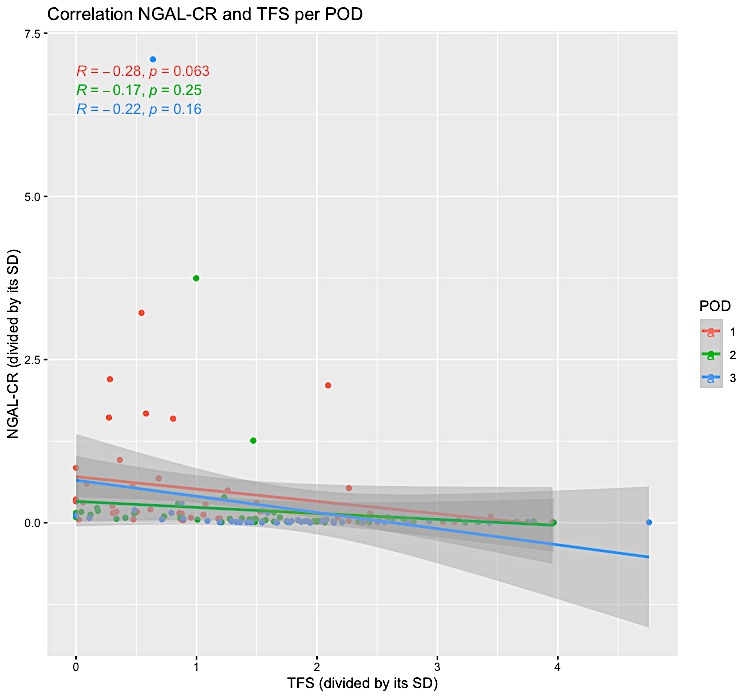

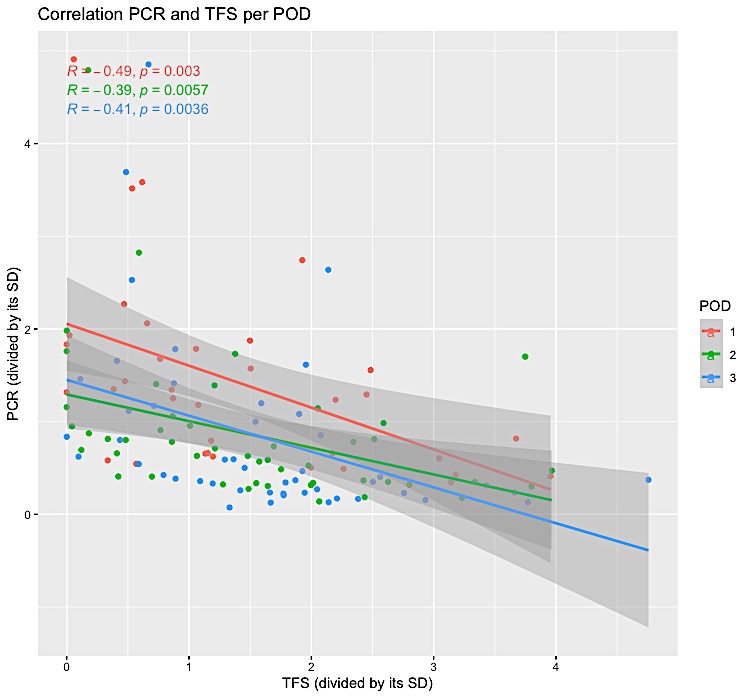

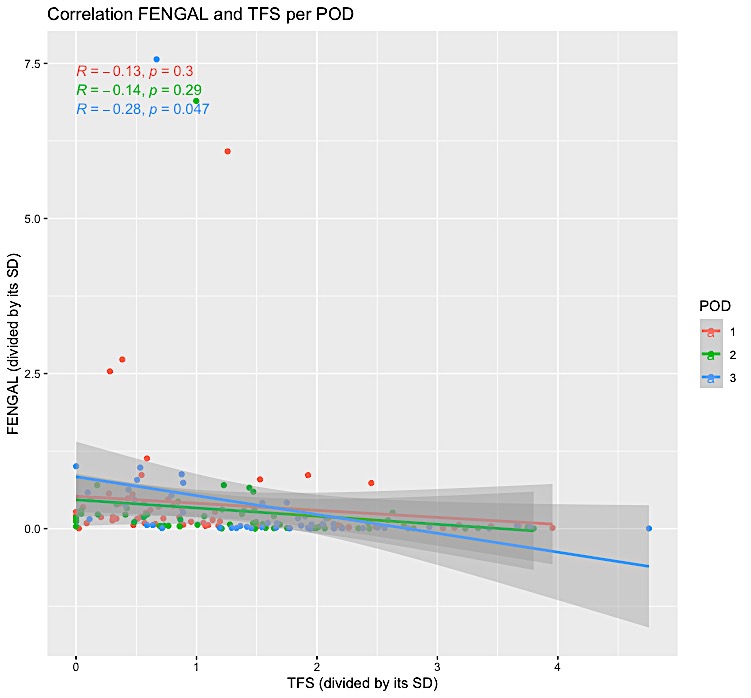

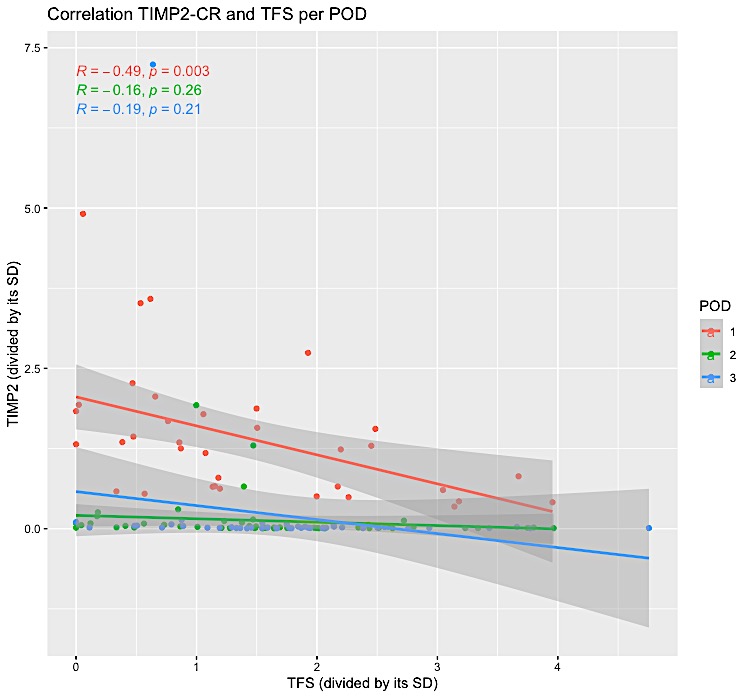


**POD**

1

4

10

**Supplemental Figure 2**. Pearson correlation plots for the correlation between TFS and creatinine-corrected urinary markers (divided by their standard deviation) on POD 1, 4 and 10 after DCD kidney transplantation. *Abbreviations*: B2M, beta-2 microglobulin; CR, creatinine ratio; DCD, donation after circulatory death; FE, fractional excretion; NGAL, neutrophil gelatinase-associated lipocalin; P-CR, protein to creatinine ratio; POD, postoperative day; R, Pearson correlation coefficient, SD, standard deviation; TIMP2, tissue inhibitor of metalloproteinases-2; TFS, tubular function slope.

TFS, divided by its SD

TFS, divided by its SD

FENGAL, divided by its SD

FEB2M, divided by its SD

**FEB2M and TFS**

B2M-CR, divided by its SD

**B2M-CR and TFS**

NGAL-CR, divided by its SD

P-CR, divided by its SD

**P-CR and TFS**

TIMP2-CR, divided by its SD

**TIMP2 and TFS**

**FENGAL and TFS**

**NGAL-CR and TFS**

**Prediction of fDGF presence**

**POD 10**

**POD 4**

**POD 1**


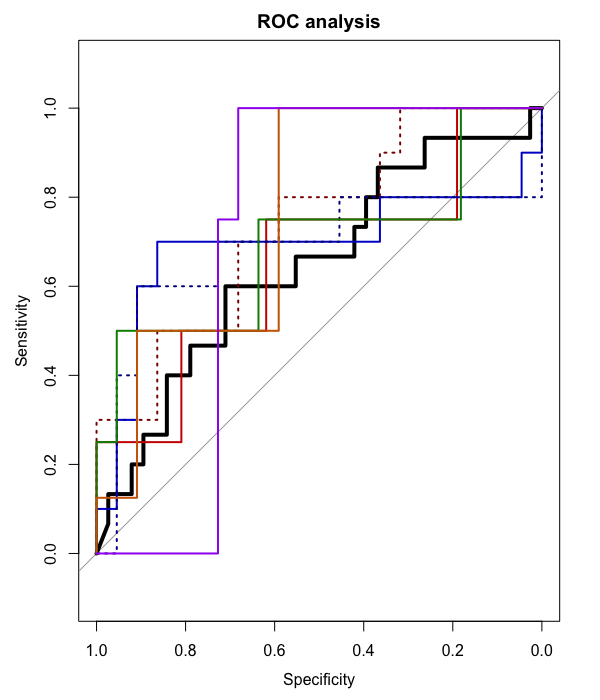

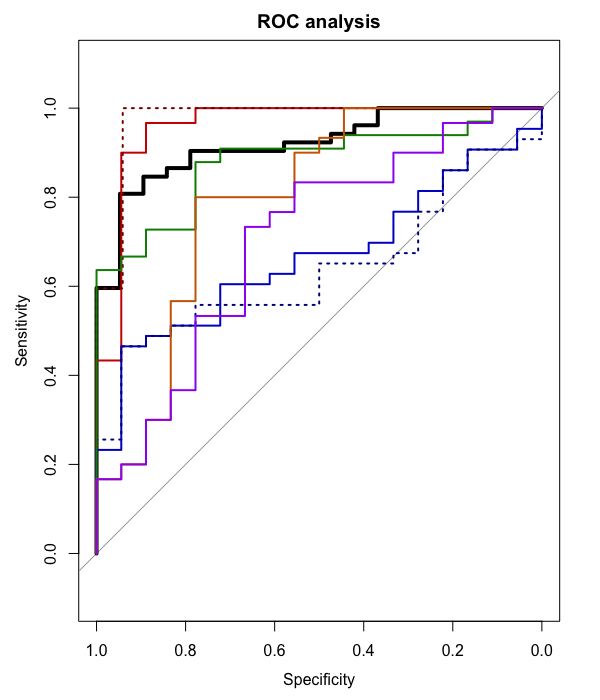

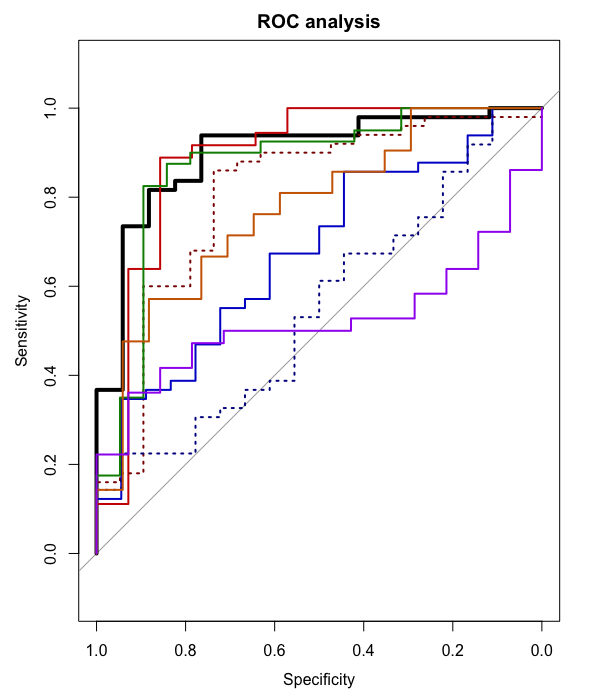


TFS 0.65

NGAL-CR 0.65

FENGAL 0.74

B2M-CR 0.69

FEB2M 0.68

TIMP2-CR 0.69

PCR 0.76

NGAL/TIMP2 0.72

TFS 0.90

NGAL-CR 0.89

FENGAL 0.81

B2M-CR 0.68

FEB2M 0.55

TIMP2-CR 0.87

PCR 0.77

NGAL/TIMP2 0.52

TFS 0.92

NGAL-CR 0.96

FENGAL 0.98

B2M-CR 0.67

FEB2M 0.64

TIMP2-CR 0.88

PCR 0.79

NGAL/TIMP2 0.70

**Legend** **AUC**

**Legend** **AUC**

**Legend** **AUC**

**Supplemental Figure 3**. ROC analysis for the prediction of fDGF presence on POD 1 and 4, and prolonged fDGF duration on POD 10 by standardized TFS and standardized creatinine-corrected urinary markers on POD 1, 4 and 10 after DCD kidney transplantation. *Abbreviations*: AUC, area under the curve; B2M, beta-2 microglobulin; CR, creatinine ratio; DCD, donation after circulatory death; fDGF, functional delayed graft function; FE, fractional excretion; NGAL, neutrophil gelatinase-associated lipocalin; P-CR, protein to creatinine ratio; POD, postoperative day; TIMP2, tissue inhibitor of metalloproteinases-2; TFS, tubular function slope.

| **Supplemental Table 4.**  Overview of missing urinary samples and measurements on POD 1, 4 and 10 among 89 recipients of DCD kidney transplantation. | | | |
| --- | --- | --- | --- |
|  | **Number available, (%)** | | |
|  | **POD 1** | **POD 4** | **POD 10** |
| *Total study population* | *89* | *89* | *89* |
| **Urine samples available** | 70 (79) | 73 (82) | 64 (72) |
| Unavailable because uVolume <500 | 12 (13) | 14 (16) | 12 (13) |
| Reason for missing unknown | 7 (8) | 2 (2) | 13 (15) |
| **Markers measured if urine available** | *70* | *73* | *64* |
| Total protein | 38 (54) | 54 (74) | 57 (89) |
| NGAL | 50 (71) | 56 (77) | 51 (80) |
| FE-NGAL | 50 (71) | 56 (77) | 51 (80) |
| B2M | 67 (96) | 72 (99) | 58 (91) |
| FE-B2M | 67 (96) | 69 (95) | 57 (89) |
| TIMP2 | 59 (84) | 60 (82) | 53 (83) |
| **Complete cases** | 25 (36) | 44 (60) | 42 (66) |
| POD 1, 4 & 10 | 16 (23) | 16 (22) | 16 (25) |
| POD 4 & 10 | - | 32 (44) | 32 (50) |
| *Abbreviations*: B2M, beta-2 microglobulin; DCD, donation after circulatory death; FE, fractional excretion; NGAL, neutrophil gelatinase-associated lipocalin; P-CR, protein to creatinine ratio; POD, postoperative day; TIMP2, tissue inhibitor of metalloproteinases-2. | | | |

| **Supplemental Table 5.** Area under the curve (95% CI) from ROC analysis for the prediction of fDGF presence on POD 4, and prolonged fDGF duration on POD 10 by standardized TFS and standardized creatinine-corrected urinary markers on POD 4 and 10 of 32 complete cases after DCD kidney transplantation. | | |
| --- | --- | --- |
|  | **AUC (95% CI)** | |
| **Urinary marker**  **divided by SD** | **fDGF** | **Prolonged fDGF** |
|  | POD 4 | POD 10 |
| TFS | 0.80 (0.63-0.96) | 0.81 (0.44-0.97) |
| NGAL-CR | 0.92 (0.81-1.00) | 0.56 (0.30-0.93) |
| FE-NGAL | 0.96 (0.88-1.00) | 0.74 (0.41-0.86) |
| B2M-CR | 0.67 (0.46-0.89) | 0.59 (0.27-0.92) |
| FE-B2M | 0.67 (0.45-0.89) | 0.67 (0.26-0.90) |
| TIMP2-CR | 0.74 (0.54-0.94) | 0.63 (0.34-0.79) |
| P-CR | 0.76 (0.57-0.94) | 0.78 (0.72-1.00) |
| NGAL/TIMP2 | 0.82 (0.66-0.98) | 0.56 (0.29-0.93) |
| *Abbreviations*: AUC, area under the curve; B2M, beta-2 microglobulin; CR, creatinine ratio; DCD, donation after circulatory death; ECC, endogenous creatinine clearance; fDGF, functional delayed graft function; FE, fractional excretion; NGAL, neutrophil gelatinase-associated lipocalin; P-CR, protein to creatinine ratio; POD, postoperative day; TIMP2, tissue inhibitor of metalloproteinases-2; TFS, tubular function slope. | | |
